# Closing the gender gap in Pakistan’s pharmaceutical workforce: evidence, insights, and a policy agenda

**DOI:** 10.1080/20523211.2025.2600240

**Published:** 2025-12-22

**Authors:** Nadia Bukhari, Bismah Nayyer, Madiha Malik, Mehr Muhammad Adeel Riaz, Madiha Sajid

**Affiliations:** a Practice and Policy, University College London, London, UK; b Population Health Sciences, King's College London, UK; c Practice and Policy, Hamdard University, Gadap Town, Pakistan; d Medicine, Faisalabad Medical University, Faisalabad, Pakistan; e Organisation Development, Department of Life Sciences, Imperial College London, London, UK

## Introduction

Gender inequity in the health workforce is a persistent drag on health system effectiveness, economic productivity, and population health (World Health Organization, 2019). Women make up most of the global health and social workforce yet remain under-represented in decision-making roles and are disproportionately affected by pay gaps, safety risks, and limited access to leadership development (Cucinotta & Vanelli, 2020; World Economic Forum, 2022). In Pakistan, these inequities are compounded by social norms, fragile workforce governance, and uneven implementation of gender-responsive policies (Khan, 2007; UCL, 2021; UHC2030, 2021). Pharmacists are pivotal for safe medicine use, chronic disease management, public health delivery, and primary care, but their roles are often under-recognised and under-utilised, which narrows opportunities for women and limits health system performance (International Pharmaceutical Federation, 2020; Legraien, 2018; WHO, 2019). This editorial synthesises baseline workforce patterns, illuminates barriers that impede women’s advancement in pharmacy, and proposes a practical, policy-oriented response. It also outlines the role of Equity Pakistan as a national platform that can catalyse gender-balanced workforce transformation.

## Underpinning evidence and approach

This editorial draws on two types of evidence. First, a national snapshot from an online survey of 130 pharmacists across different sectors and career stages assessed perceptions on workplace environment, mentorship, career planning, and equity. The instrument showed robust reliability (Cronbach’s α = 0.76), and ethical clearance was obtained from Hamdard University. Second, a narrative synthesis of 47 sources includes workforce studies, regulatory analyses, and comparative policy examples. Key among these is a commentary by Bukhari et al. (2020) highlighting the urgent need for gender equity in the pharmaceutical workforce during the COVID-19 pandemic and the FIP National Level Intelligence Report: Preliminary Country Analysis – Pakistan (2023). Bukhari et al. (2023) which examines systemic workforce challenges including regulatory gaps, training deficiencies, and pharmacy service underutilisation. This builds on wider scholarship that documents barriers to women’s advancement in Pakistan’s workforce (Ali, 2013; Ali & Knox, 2008; Center for Global Development, 2022; Khan, 2010; Modern Diplomacy, 2022) and comparative studies that link gender diversity to stronger innovation and organisational outcomes (Dai et al., 2019).

## Key insights from the evidence base

Representation in the surveyed workforce was close to parity, with women comprising 49.2% of respondents, but progression to leadership was rare; only 7.6% reported holding leadership roles. This mismatch between representation and progression suggests structural bottlenecks at key transition points, including first-line supervisory roles and mid-career management positions. The sample was dominated by early-career professionals, which underscores the importance of retention and progression if Pakistan is to build a robust leadership pipeline (Global Institute for Women’s Leadership, 2025; Legraien, 2018).

Mentorship and sponsorship emerged as conspicuous gaps. Only 24.6% of respondents reported access to a mentor who actively supported their career progression, and fewer than a quarter described substantive coaching conversations with supervisors. Without structured mentorship and committed sponsorship, promising professionals lack visibility, advocacy, and the stretch opportunities that determine promotion velocity, a pattern that mirrors findings in the international literature (Gubbins et al., 2015; Hewlett & Rashid, 2015; Janzen et al., 2007).

The workplace climate appeared mixed. Many pharmacists described comfort working in mixed-gender teams and noted the presence of positive role models, yet a substantial proportion believed female colleagues encountered additional challenges at work. Reports from the wider policy environment on harassment, safety, and inconsistent enforcement provide plausible mechanisms for attrition and stalled careers (International Monetary Fund, 2019; Pakistan Bureau of Statistics, 2022; Punjab Commission on the Status of Women, 2021; World Bank, 2022). In this context, professional development was the most frequently requested remedy. Respondents prioritised greater access to training, leadership preparation, international exposure, transparent decision-making, and stronger professional networks, framing development not as a perk but as a precondition for fair competition (Friedrich-Ebert-Stiftung, 2020; Khan, 2007; Moazam & Shekhani, 2018).

Systemic constraints in Pakistan’s pharmacy ecosystem amplify these gendered frictions. The pharmacist-to-population ratio remains far below global benchmarks, regulatory enforcement is uneven, and clinical training opportunities are limited (International Pharmaceutical Federation, 2020). When organisational slack is low and regulatory structures are weak, those already negotiating social expectations (often women), face steeper drop-off points (Hafeez, 1991; Mumtaz & Shaheed, 1987). Economic analyses indicate that narrowing gender participation gaps would lift GDP and improve fiscal resilience while expanding the supply of skilled health workers (International Monetary Fund, 2020; Mahmood, 2011; UN Women Pakistan, 2023). The evidence therefore supports a dual rationale: gender equity is both the right course and the efficient one (Sathar & Kazi, 2000).

## Discussion and policy implications

A credible response begins with making equity non-negotiable through regulation and enforcement. Transparent pay bands, routine gender pay audits, and timely corrective action are essential to establish trust and to signal that meritocracy is more than rhetoric (International Labour Organization, 2018; World Economic Forum, 2022). Safety and dignity at work require visible enforcement of anti-harassment laws, confidential reporting mechanisms, and consistent organisational consequences (Government of Pakistan, 2020; Pakistan Bureau of Statistics, 2022). Without these foundations, even the most sophisticated leadership programmes will fail to retain women in the workforce.

Leadership pipelines and mentorship must be designed and executed at scale. National and institutional programmes should pair early-career women with senior leaders across practice settings, academia, and industry, with participation tracked as a leadership key performance indicator. Leadership preparation should extend beyond short courses to include fellowships, secondments, and stretch assignments, particularly in rural or underserved areas where the need for clinical pharmacy leadership is greatest. These interventions shift opportunities from informal networks to structured, accountable pathways, reducing the reliance on ad hoc sponsorship (Global Institute for Women’s Leadership, 2025; Khan, 2010; Khan & Naqvi, 2020).

Education, continuous professional development, and role utilisation must be modernised in tandem. Curriculum reform that expands hands-on clinical training, structured internships and residencies, and competencies in pharmacovigilance, medicines optimisation, and prescribing readiness will help align the workforce with contemporary care models (International Pharmaceutical Federation, 2020). Making CPD a requirement for re-licensure, supported by a national digital registry that documents portfolios and completion, creates clear expectations for skill renewal and provides regulators with actionable data. Including leadership, management, and patient-safety modules within CPD will normalise competencies that are often assumed rather than taught (Ali, 2013; International Labour Organization, 2020).

Practical barriers that disproportionately affect women demand pragmatic solutions. Flexible scheduling, part-time tracks, predictable rosters, and reliable return-to-work policies after childbirth increase retention and reduce the penalty for caregiving (Moazam & Shekhani, 2018; Modern Diplomacy, 2022). Safer commutes, appropriate facility design, and attention to basic amenities, such as lactation rooms and secure rest areas, are often inexpensive but symbolically powerful investments. Where community and retail settings involve night shifts or remote locations, partnerships between employers and local authorities can strengthen security and reduce attrition (Center for Global Development, 2022; Sathar & Kazi, 2000).

Entrepreneurship offers an additional level for equity. Targeted incentives, such as preferential access to credit, tax relief, and technical assistance can support women-owned pharmacies and digital health ventures, widen decision-making representation, and create visible role models for the next generation. Transparent promotion systems inside organisations, with posted criteria, diverse panels, and accessible appeal mechanisms, reduce the opacity that frequently masks bias and slows women’s advancement (Government of Pakistan, 2020; Khan, 2007; Mahmood, 2011; Roomi & Parrott, 2008).

Finally, progress depends on workforce intelligence and accountability. A unified digital register of pharmacists, disaggregated by gender, sector, and location, would support planning, placements, return-to-practice initiatives, and scholarship targeting. Public equity dashboards at institutional and provincial levels would create shared visibility and healthy pressure to improve. Linking a portion of public funding, accreditation, or recognition to demonstrable equity gains would align incentives without adding undue administrative burden (Ali & Knox, 2008; Friedrich-Ebert-Stiftung, 2020; World Bank, 2022).

## Equity Pakistan: a platform to drive change

Equity Pakistan, launched in 2021, occupies a strategic position at the intersection of research, policy, and practice (UCL, 2021). Through collaborations with the UCL School of Pharmacy and the Hamdard Institute of Pharmaceutical Sciences, and with support from FIP, WHO, and other partners, the platform is well placed to scale mentorship and leadership pipelines, advance policy harmonisation on pay, safety, and flexibility, and convene key stakeholders to align incentives and track progress. This aligns with wider evidence highlighting the importance of coordinated, multi-stakeholder initiatives in advancing gender equity in the workforce (Ali, 2013; Ali & Knox, 2008; Global Institute for Women’s Leadership, 2025).

By developing practical toolkits, such as model anti-harassment policies, pay audit templates, and mentorship frameworks and by championing data systems, including a national CPD and competency registry, Equity Pakistan can provide a consistent focal point for action across provinces and sectors. Its role as a national hub ensures continuity, standard setting, and shared accountability, which are frequently missing in fragmented reform efforts (Center for Global Development, 2022; Modern Diplomacy, 2022).

## From parity to power: unlocking pharmacy’s future

Pakistan’s pharmacy workforce stands at a pivotal moment. The talent base is present, the health system need is pronounced, and the economic case for equity is compelling. Yet women remain under-represented in leadership, hampered by thin mentorship, uneven protections, and limited access to development pathways. The remedy is practical and well supported by evidence: enforce pay equity and safety; professionalise mentorship and leadership pipelines; modernise education and CPD; remove everyday barriers that disproportionately affect women; catalyse entrepreneurship; and measure progress with transparent data.

Success will require coordinated effort by policymakers, regulators, employers, academic leaders, and professional bodies, anchored by a national platform capable of sustaining momentum. Gender equity is not a peripheral concern but a central element of workforce policy. Advancing it will strengthen pharmaceutical care, improve patient outcomes, and build a resilient health system.

Empowering women in pharmacy is therefore both an ethical obligation and a strategy to unlock the full potential of Pakistan’s healthcare future.

## Authors’ contributions

All authors participated in the manuscript's study design, conceptualisation, drafting, and revision. All authors have read and approved the final text of the manuscript.

## Ethics approval and consent to participate

Not applicable.

## Consent for publication

All authors approved the manuscript.
